# Retinal Image Enhancement Using Robust Inverse Diffusion Equation and Self-Similarity Filtering

**DOI:** 10.1371/journal.pone.0158480

**Published:** 2016-07-07

**Authors:** Lu Wang, Guohua Liu, Shujun Fu, Lingzhong Xu, Kun Zhao, Caiming Zhang

**Affiliations:** 1 School of Public Health, Shandong University, Jinan 250012, China; 2 Department of Ophthalmology, Qilu Children’s Hospital of Shandong University, Jinan 250022, China; 3 School of Mathematics, Shandong University, Jinan 250100, China; 4 Department of Medical Imaging, The Second Hospital of Shandong University, Jinan 250033, China; 5 School of Computer Science and Technology, Shandong University of Finance and Economics, Jinan 250061, China; 6 School of Computer Science and Technology, Shandong University, Jinan 250101, China; Beijing University of Technology, CHINA

## Abstract

As a common ocular complication for diabetic patients, diabetic retinopathy has become an important public health problem in the world. Early diagnosis and early treatment with the help of fundus imaging technology is an effective control method. In this paper, a robust inverse diffusion equation combining a self-similarity filtering is presented to detect and evaluate diabetic retinopathy using retinal image enhancement. A flux corrected transport technique is used to control diffusion flux adaptively, which eliminates overshoots inherent in the Laplacian operation. Feature preserving denoising by the self-similarity filtering ensures a robust enhancement of noisy and blurry retinal images. Experimental results demonstrate that this algorithm can enhance important details of retinal image data effectively, affording an opportunity for better medical interpretation and subsequent processing.

## Introduction

With the development of economy and the aging population, people’s visual impairments has become a major public health problem all over the world. All kinds of ophthalmic diseases causing visual defects not only increase the burden of public health care system, more importantly, they also pose serious threats to social and economic activities [1, 2]. Among them, as one of main blinding eye diseases, diabetic retinopathy is the most common ocular complication in diabetic patients, which includes a series of typical lesions with retinal microvascular and neuron damages caused by sugar metabolic abnormalities. It is a chronic and progressive blinding fundus disease which can be characterized by such clinical features as decreased vision, fundus bleeding and exudation, macular edema and hyperplastic lesions [3]. The overall prevalence of diabetic retinopathy was 34.6% according to a meta-analysis of 35 international renowned epidemiological studies (22896 cases of diabetes) of the world [4]. The fact of high prevalence, high blindness rate, high fashion trend, high social and economic burden, and low cognition rate makes things worse [2].

Early diagnosis and early treatment is an effective method for the control of diabetic retinopathy [5, 6]. Fundus imaging by digital fundus camera is a standard diagnostic mode in ophthalmology, which captures the intensity of light reflected from the retinal surface in three different wavelength ranges [7, 8]. By reason of imaging mechanism and system of fundus retina imaging itself, and the disturbance of various noise in image formation process, one often obtain noisy and blurry retinal image with nonuniform and distorted illumination, which is difficult to interpret medically and to process subsequently [7, 9–11]. Thus, it is indispensable to remove noise and disturbances, to improve signal-to-noise rate of image, to adjust image contrast and to enhance vessels and fine details of retinal image data [9, 12, 13]. By above image preprocessing, useful information in retinal image is highlighted, while useless one is weakened or removed, to make the result more suitable to clinical diagnosis and treatment [3, 7, 9, 14–17].

Many different methods have been put forth for retinal image denoising and enhancement [7, 9, 18, 19], such as the Gamma transformation [18], histogram equalization [20, 21], sharpening by the Laplacian operation [22], filtering methods in transformation fields [19], variational methods and partial differential equations (PDEs) [13, 23, 24]. However, one of major challenges faced by these methods is, how to avoid enhancing noise, producing overshoot artifacts around edges, and erasing fine details in enhanced images [23, 25, 26].

Important medical information of a retinal image lies in its retinal vessel network and local fine details. In order to accurately detect and evaluate diabetic retinopathy as soon as possible, it is very crucial to properly enhance possible retinal pathological features such as microaneurysm, bleeding and exudating spots. In this paper, a robust inverse diffusion equation is presented, which combines a powerful self-similarity filtering [27, 28] for detail preserving image denoising. A flux corrected transport (FCT) technique [25, 29] is used to control diffusion flux adaptively, which effectively eliminates overshoots inherent in the Laplacian operation. The proposed method extends our previous work [25] to enhance noisy images while avoiding annoying overshoots and noise magnification.

We organize this paper as follows. In Section **II**, related image enhancement methods by the Gamma transformation, the shock computing and the self-similarity filtering are introduced. In Section **III**, the robust inverse diffusion equation is built to enhance noisy and blurry retinal image data, where the flux corrected transport technique is elaborated in a subsequential process including three main steps. In Section **IV**, experiments on retinal images with typical diabetic retinopathy are carried out to verify the effectiveness of the proposed algorithm. Finally, conclusions and future work are included to end this paper in section **V**.

## Related image enhancement methods (II)

Intensity transformation is the simplest technique in image enhancement through mapping a pixel value r into a pixel value z, among which the Gamma (power-law) transformation is one of basic transformation functions [18]. It is defined as

$$\begin{array}{c} z=br^{\gamma }, \end{array}$$ (1)

where, *b* and *γ* are positive constants. The Gamma transformation with fractional values of *γ* can map a narrow range of input values into a wider range of output values. In a variety of devices the Gamma transformation is used to appropriately enhance image contrast and details for image capture, printing and display.

In the past decades there has been an increasing research on partial differential equations (PDEs) in image enhancement [23, 30–32]. A great deal of successful applications of nonlinear evolving PDEs in image enhancement can mainly be attributed to their two basic characteristics: local operation and iterative processing. Osher and Rudin introduced a novel image sharpening technique, called the shock filter (SF) [30], which simulates the shock wave calculation in computational fluid mechanics:

$$\begin{array}{c} \frac{\partial u}{\partial t}=-sign\left(u_{NN}\right)\left|\nabla u\right|,\;u_{NN}=\frac{1}{{\left|\nabla u\right|}^{2}}\left(u_{x}^{2}u_{xx}+2u_{x}u_{y}u_{xy}+u_{y}^{2}u_{yy}\right), \end{array}$$ (2)

where *sign* is a sign function, ∇ is a gradient operator, and *u*_*NN*_ is the second directional derivative of image along local normal direction to isophote line. It detects an image edge using the zero-crossing of *u*_*NN*_, where a shock is formed at a speed of the gradient magnitude |∇*u*|.

Considering image noise in the estimation of edges, Alvarez and Mazorra added a smoothing kernel and coupled the anisotropic diffusion with the shock filter (ADSF) [31, 32] for noise elimination and edge sharpening:

$$\begin{array}{c} \frac{\partial u}{\partial t}=-sign\left(G_{\sigma }*u_{NN}\right)\left|\nabla u\right|+cu_{TT},\;u_{TT}=\Delta u-u_{NN}, \end{array}$$ (3)

where Δ is a Laplacian operator, *G*_*σ*_ is a Gaussian kernel with standard deviation *σ*, *u*_*TT*_ is the second directional derivative of image along local tangent direction, and *c* is a constant to balance the anisotropic diffusion and the shock filtering.

On the other hand, in order to effectively denoise images while preserving image details, a powerful non-local means algorithm was proposed by Buades et al. [27], which fully utilizes the big redundancy and the self-similarity of natural images in the photometric range. The discrete expression of the self-similarity filtering (SSF) algorithm is as follows. Let *u* be a noisy image defined in a discrete grid $\Omega \subset R^{2}$. The denoised intensity at the pixel (*i*, *j*) is expressed by

$$\begin{array}{c} SSF\left(u_{ij}\right)=\sum_{(m,n)\in \Omega }w_{ij}\left(m,n\right)u_{mn}/\sum_{(m,n)\in \Omega }w_{ij}\left(m,n\right), \end{array}$$ (4)

where *w*_*ij*_(*m*, *n*) is an average weight which is determined by the similarity between the pixels (*i*, *j*) and (*m*, *n*), and is adopted as

$$\begin{array}{c} w_{ij}\left(m,n\right)=\exp\left\{-||u\left(N_{ij}\right)-u\left(N_{mn}\right){||}_{2,a}^{2}/h^{2}\right\}, \end{array}$$ (5)

where *N*_*ij*_ and *N*_*mn*_ are similarity windows of size (2*s* + 1) × (2*s* + 1) centered at pixels (*i*, *j*) and (*m*, *n*), respectively. The term *u*(*N*_*ij*_) denotes an image patch restricted in the similarity window *N*_*ij*_. The notation || ⋅ ||_2,*a*_ denotes a Gaussian weighted Euclidean distance between two image patches, where *a* is the standard deviation of the Gaussian function. The parameter *h* denotes a smoothing factor that controls the decay of the exponential function in the Eq (5). To reduce the computational burden and to improve the efficiency of the SSF filtering, the search window is always restricted to a proper local neighborhood (2*f* + 1) × (2*f* + 1) in Ω. The denominator in the Eq (4) is a normalizing factor.

## Robust inverse diffusion equation (III)

As special inverse diffusion processing [23], although the shock computing [30–33] can effectively sharpen image edges and remove image noise, there are some inherent weaknesses for it to enhance retinal image. Firstly, for noisy and blurry retinal image data, it is difficult to estimate its local tangential and normal directions; and for finer details these directions are difficult to define and estimate. Secondly, in order to enhance tiny lesions important for the detection of diabetic retinopathy, where the value of image gradient is very small, it is improper for the shock computing at a speed of the gradient magnitude. Thirdly, the Gaussian and tangential smoothings in the shock computing easily erase important details when removing noise and smoothing the second directional derivative. Finally, unnatural artifacts may be produced around image edges where shocks are formed by the shock computing [23]. These defects will be compared and shown in following experiments of enhancing retinal images.

In order to overcome above difficulties, we present the following robust inverse diffusion (RID) equation:

$$\begin{array}{c} \frac{\partial u}{\partial t}=-\left|\nabla u\right|\Delta u, \end{array}$$ (6)

When solving numerically a nonlinear inverse diffusion equation like Eq (6) using a difference scheme, it must be discretized carefully because it is an instable process. Otherwise, numerical blowing up will appear inevitably. A strategy is to try to stop from numerical fluctuations before they appear, which is based on the Total Variation Diminishing (TVD) and nonlinear limiters [32, 34]. The main idea of above flux corrected transport technique is to use a limiter function to control the change of the numerical solution by a nonlinear way, and the corresponding schemes satisfy the TVD condition and consequently eliminate above disadvantage effects.

**Fig 1:**
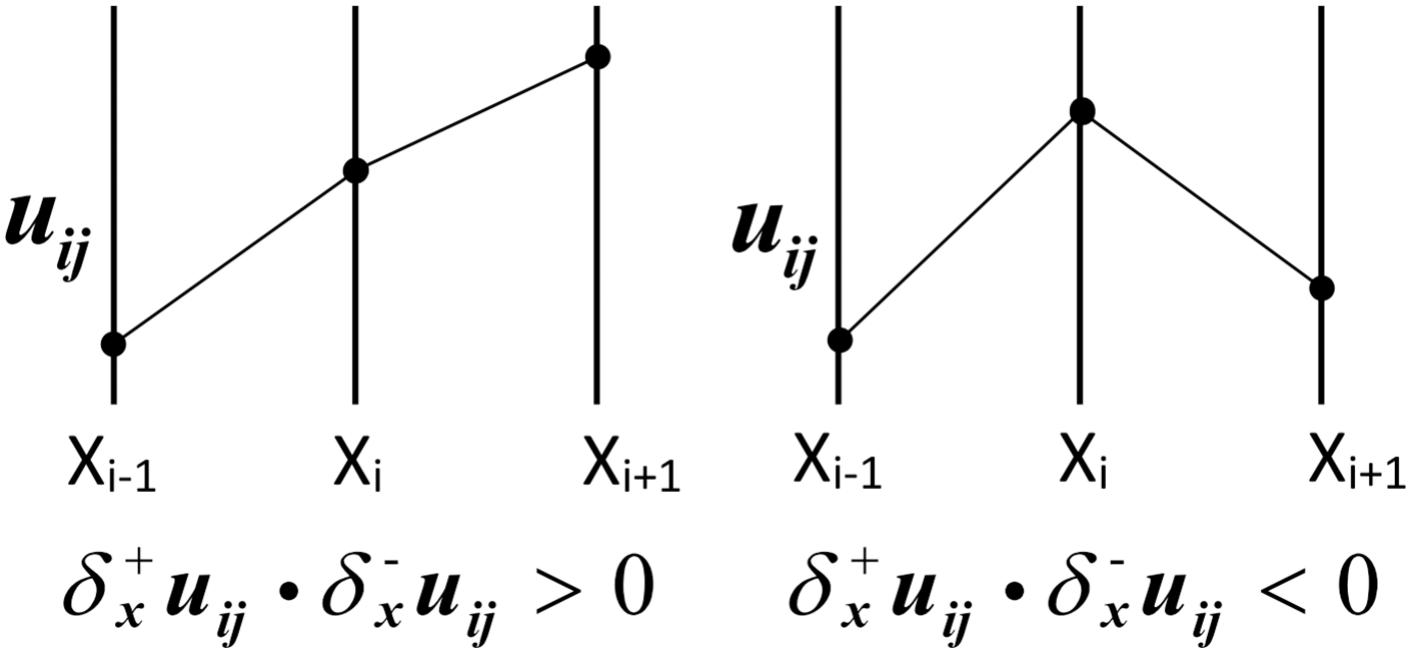
Illustration of limiter function *M*(*p*, *q*): two situations for forward and backward difference schemes. *M* function stops Eq (6) from numerical fluctuations (overshoots) along data points (*X*_*i*−1_, *X*_*i*_, *X*_*i*+1_).

An explicit Euler method with central difference scheme is used to approximate the Eq (6) except the gradient term. Below we detail a approach to it numerically. On the image grid Ω, the approximate solution is to satisfy:

$$\begin{array}{c} u_{ij}^{k}\approx u\left(il,jl,k\Delta t\right),\;i,j,k\in Z^{+}, \end{array}$$ (7)

where *l* and Δ*t* are spatial and temporal steps. Let *l* = 1, and $\delta^{+}u_{ij}^{k}$ and $\delta^{-}u_{ij}^{k}$ are forward and backward difference schemes of $u_{ij}^{k}$, respectively. For example, along the *x* direction, $\delta_{x}^{+}u_{ij}^{k}=u_{(i+1)j}^{k}-u_{ij}^{k}$, $\delta_{x}^{-}u_{ij}^{k}=u_{ij}^{k}-u_{(i-1)j}^{k}$; the case is similar along the *y* direction. A limiter function *M*(*p*, *q*) is used to approximate the gradient term (see Fig 1):

$$\begin{array}{c} {\left|\nabla u\right|}_{ij}^{k}=min\left\{M\left(\delta_{x}^{+}u_{ij}^{k},\delta_{x}^{-}u_{ij}^{k}\right),M\left(\delta_{y}^{+}u_{ij}^{k},\delta_{y}^{-}u_{ij}^{k}\right)\right\}, \end{array}$$ (8)

where

$$\begin{array}{c} M\left(p,q\right)=\left\{\begin{array}{cc} \lambda , & pq>0\\ 0, & pq\leq 0. \end{array}\right. \end{array}$$ (9)

Here, *λ* is a constant to guarantee that tiny important details can also be enhanced effectively regardless of its small gradient magnitude. After the numerical discretization of Eq (6), it can be considered as an enhancement process by an iterative constrained Laplacian operation [25].

**Fig 2:**
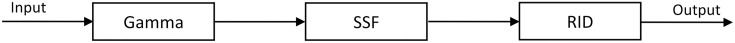
Flow chart of our proposed algorithm. A degraded image is enhanced through three steps in sequence: Gamma manipulation (Gamma), self-similarity filtering (SSF) and robust inverse diffusion (RID), respectively.

In order to improve the contrast of retinal image for the detection of tiny lesions, a Gamma transformation [18] is first used to enhance the image within proper gray levels. Then, the powerful self-similarity filtering [27] is employed to remove image noise, especially in the regions of interest (ROI). Finally, the proposed robust inverse diffusion is carried out to further sharpen important details of retinal image while avoiding overshoot artifacts. A whole flow chart of our proposed algorithm is shown in Fig 2.

## Experimental results and analyses (IV)

Although retinal images can be represented in many color spaces (RGB, HSI, HSV, etc.), the selection of them highly depends on the application. In this paper, a retinal image enhancement algorithm is designed to help physicians in their task of early diagnose of retinopathy, and therefore the selected space must be as close as possible to human perception [35]. A well-established agreement is that the green channel in the RGB color space provides more blood vessel structural information and is less subject to non-uniform illumination, while the HSV color space does not preserve the fidelity of retinal images [35–37]. Because green light is absorbed by the blood and reflected by the retinal pigment epithelium, providing a good contrast for visualizing retinal vascular network, bleeding and exudation, we routinely extract and enhance the green channel (in the gray range of [0, 1]) from a RGB color fundus photograph [7].

**Fig 3:**
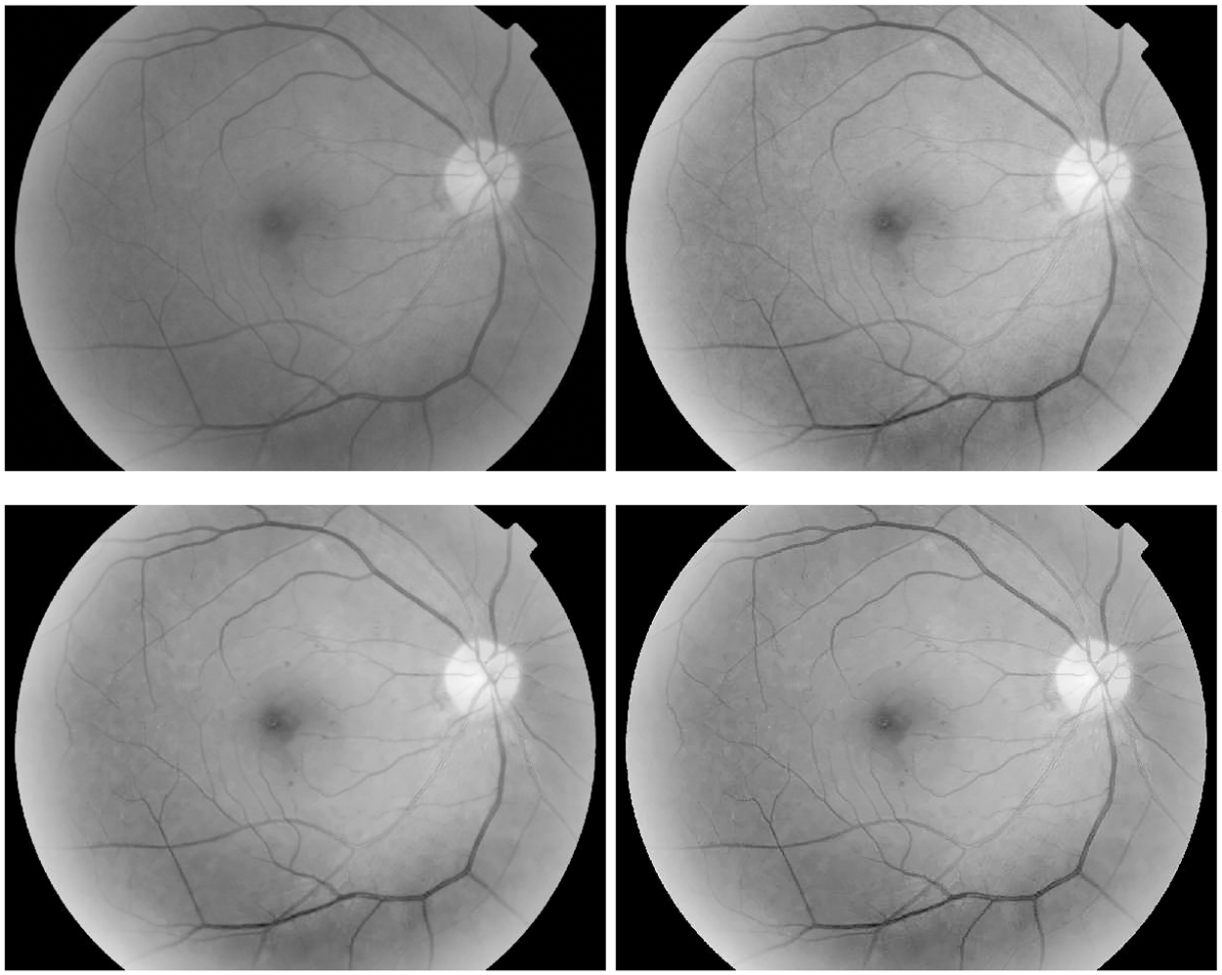
Retinal image enhancement for detection of microaneurysms in diabetic retinopathy (from top-left to bottom-right): original image, results by Gamma manipulation, self-similarity filtering and robust inverse diffusion, successively.

In Fig 3, a retinal image with tiny microaneurysms of size 465 × 600 is enhanced for the early detection of diabetic retinopathy. Through sequential processings of the Gamma manipulation (*γ* = 0.6), the self-similarity filtering (*h* = 0.01, *f* = 5, *s* = 3) and the robust inverse diffusion (*λ* = 0.6, Δ*t* = 0.15, *k* = 7), tiny microaneurysms and microvasculature are shown clearly due to image contrast improvement and noise removal. Moreover, our method produces fewer overshoot artifacts while avoiding noise magnification.

**Fig 4:**
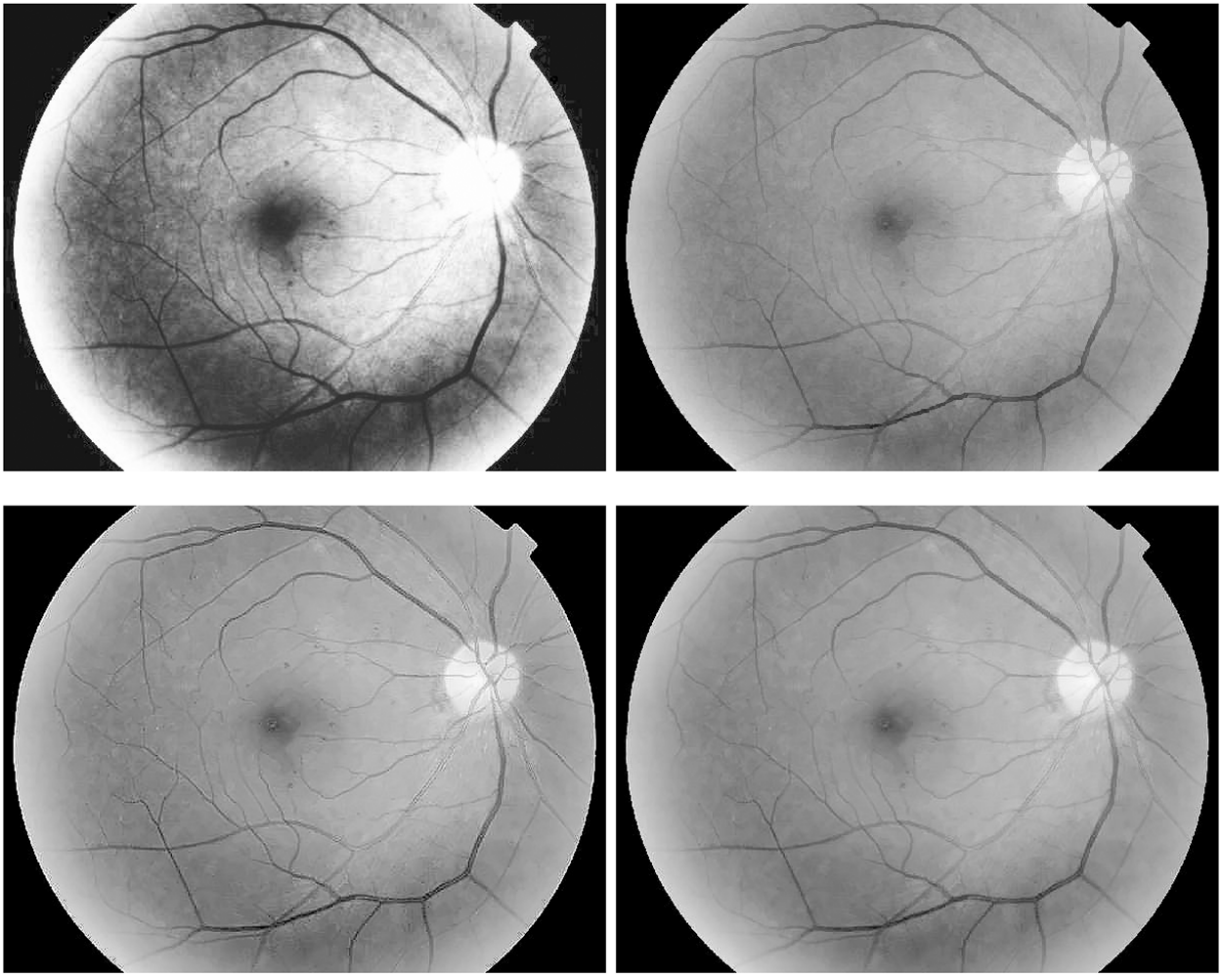
Retinal image enhancement for detection of microaneurysms in diabetic retinopathy (from top-left to bottom-right): results by histogram equalization, ADSF filtering after Gamma manipulation, Laplace operation after Gamma manipulation and self-similarity filtering, and robust inverse diffusion, respectively.

**Fig 5:**
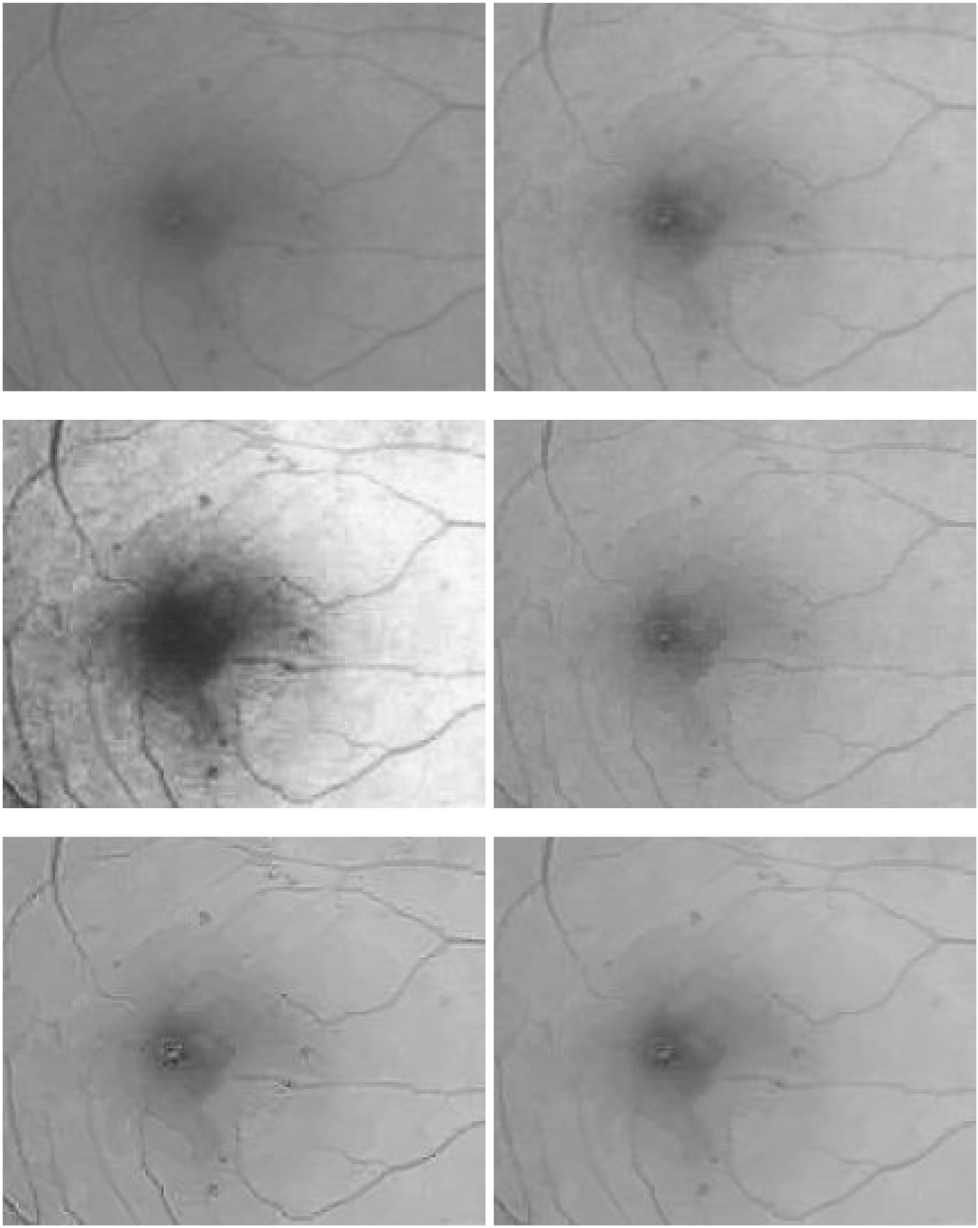
Zoomed parts of enhanced retinal images for microaneurysm detection (from top-left to bottom-right): original image, results by Gamma manipulation, histogram equalization, ADSF filtering after Gamma manipulation, Laplace operation after Gamma manipulation and self-similarity filtering, and robust inverse diffusion, respectively.

A further comparison is carried out with the histogram equalization [18], the ADSF filtering (*c* = 0.2, Δ*t* = 0.5, *n* = 10) after the Gamma manipulation, and the Laplacian operation after the Gamma manipulation and the self-similarity filtering in Figs 4 and 5, where enhancement results and their zoomed local parts are shown when enhancing the macular area by these methods. The tiny microaneurysms are not too clear in the original image shown in Fig 3. The Gamma transformation improves image contrasts on the whole, but fine spots and details remain blurry without being highlighted compared with their surrounding regions. Although the histogram equalization can enhance image contrasts to some extent, it produces nonuniform illumination distribution and noise magnification concealing some fine details. The noise magnification and artifacts (overshoots and halos) from the over-enhancing by the Laplacian operation and the ADSF filtering can be obviously observed. Moreover, the numerical blowing up will quickly come out for the multiple Laplacian operations [25], especially at bigger gradients around image edges. Only by the proposed method are tiny microaneurysms clearly shown while avoiding the artifacts and noise magnification.

**Fig 6:**
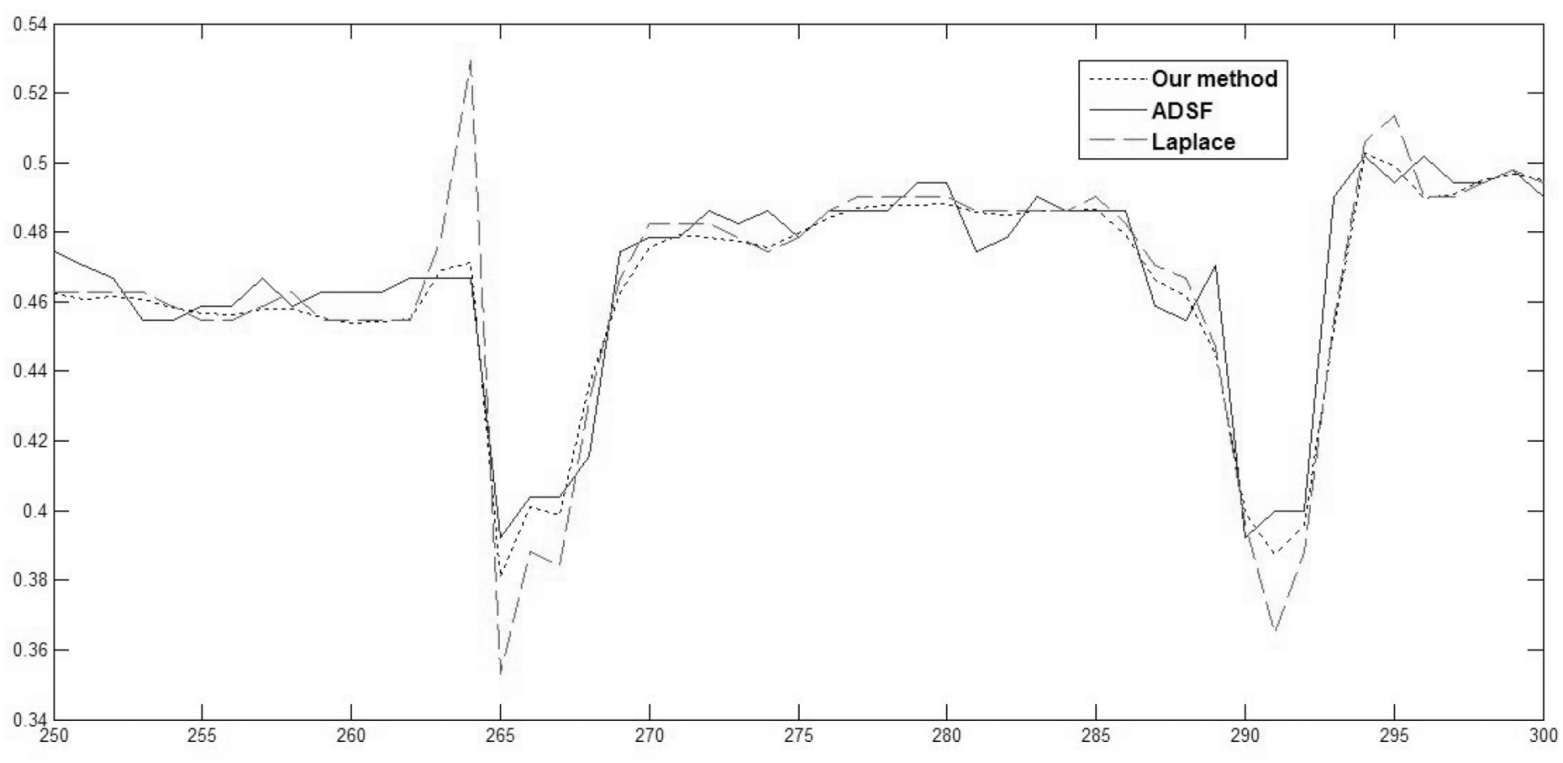
Retinal image enhancement: local comparison of profiles (350th row, 250-300 columns) of enhanced images by robust inverse diffusion, ADSF filtering and Laplacian operation, respectively.

In order to observe enhancement effects by these methods more clearly, local profiles (350th row, 250-300 columns) of different results are shown in Fig 6. One can see that, the Laplace operation produces overshoots and halos around two vessels. Because smaller *u*_*NN*_ will also be enhanced indiscriminately for shock computing, the ADSF filtering over enhances image differences and leads to annoying artifacts and false edges in flat areas of image [23]. The proposed method does not produce annoying artifacts own to its proper constrained enhancement, providing a chance to early detect the diabetic retinopathy faithfully by image enhancement.

**Fig 7:**
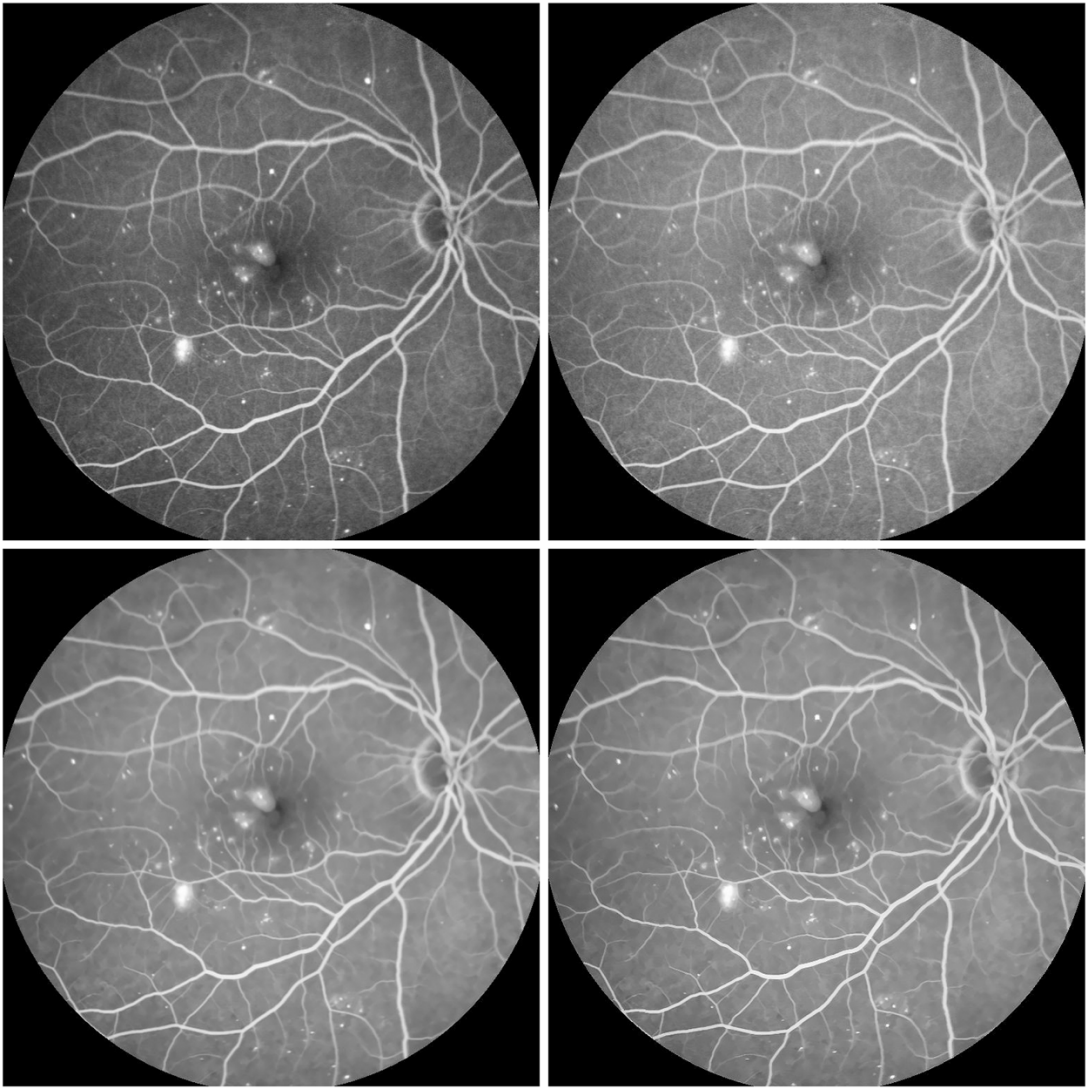
Retinal image enhancement for detection of soft exudations in diabetic retinopathy (from top-left to bottom-right): original image, results by Gamma manipulation, self-similarity filtering and robust inverse diffusion, respectively.

Next, in Fig 7, a retinal image with soft exudations of size 768 × 768 is enhanced to verify the proposed robust inverse diffusion for highlighting important medical features such as retinal vascular networks and local fine details. Through sequential processings of the Gamma manipulation (*γ* = 0.6), the self-similarity filtering (*h* = 0.03, *f* = 5, *s* = 3) and the robust inverse diffusion (*λ* = 0.5, Δ*t* = 0.15, *k* = 7), one can see that, the proposed method removes noise effectively and preserves important image details. At the same time, vascular networks and exudative spots are shown more clearly while producing no artifacts.

**Fig 8:**
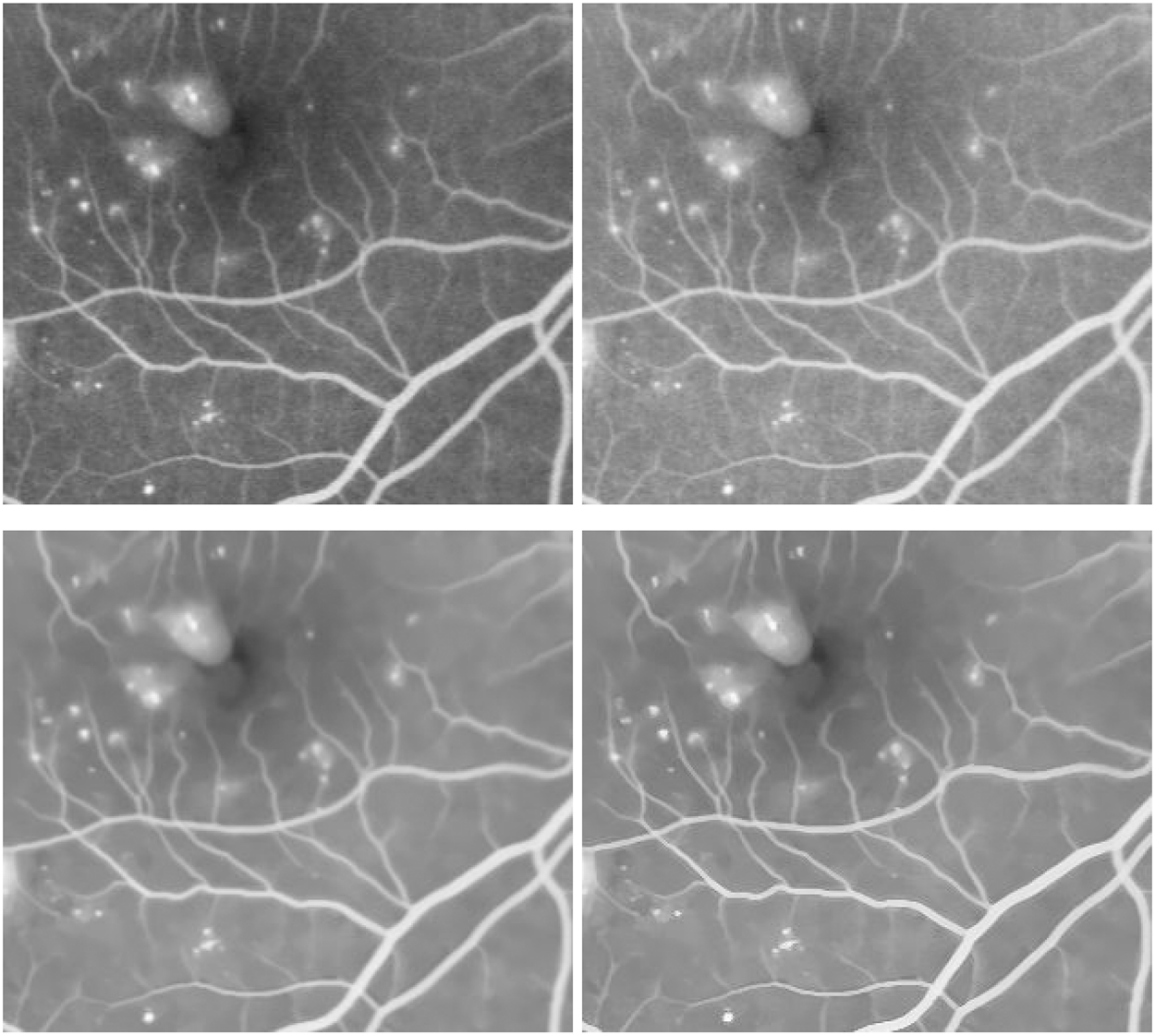
Zoomed parts of enhanced retinal images for detection of soft exudations (from top-left to bottom-right): original image, results by Gamma manipulation, self-similarity filtering and robust inverse diffusion, respectively.

In Fig 8, zoomed local parts of results by the proposed method are shown. Obviously, through sequential processings in three steps the degraded retinal image is greatly enhanced: image contrasts are improved, image noise is removed, and overshoots and halos are not produced, which further verify the advantages of the proposed method.

**Fig 9:**
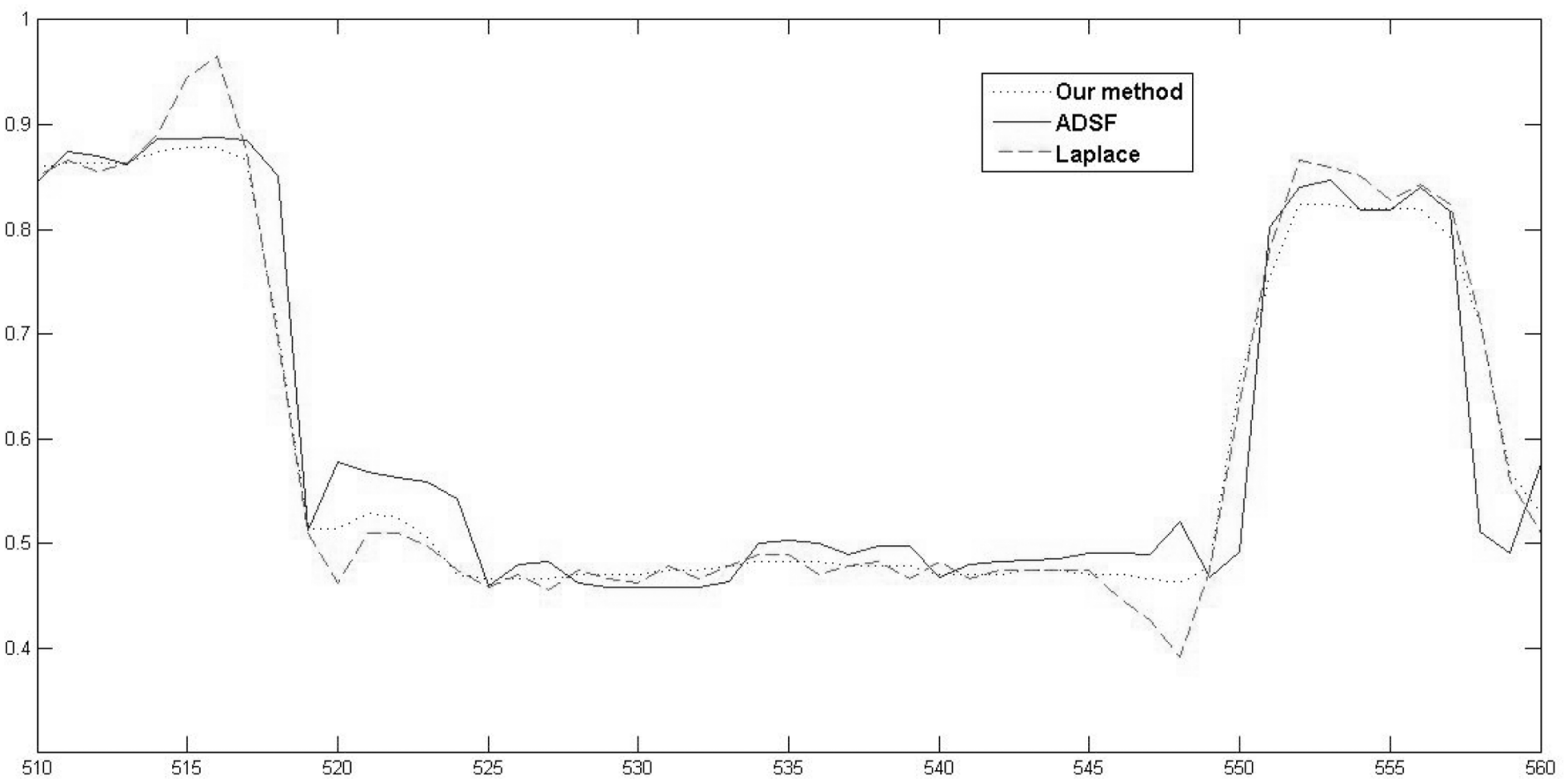
Retinal image enhancement: local comparison of profiles (350th row, 250-300 columns) of enhanced images by robust inverse diffusion, ADSF filtering and Laplacian operation, respectively.

Local profiles (500th row, 510-560 columns) of different enhancement results are also shown in Fig 9. Similarly, one can see that, both the Laplace operation and the ADSF filtering produce overshoots and halos around two vessels. The proposed method does not produce annoying artifacts, ensuring a retinal image enhancement as faithful as possible.

**Fig 10:**
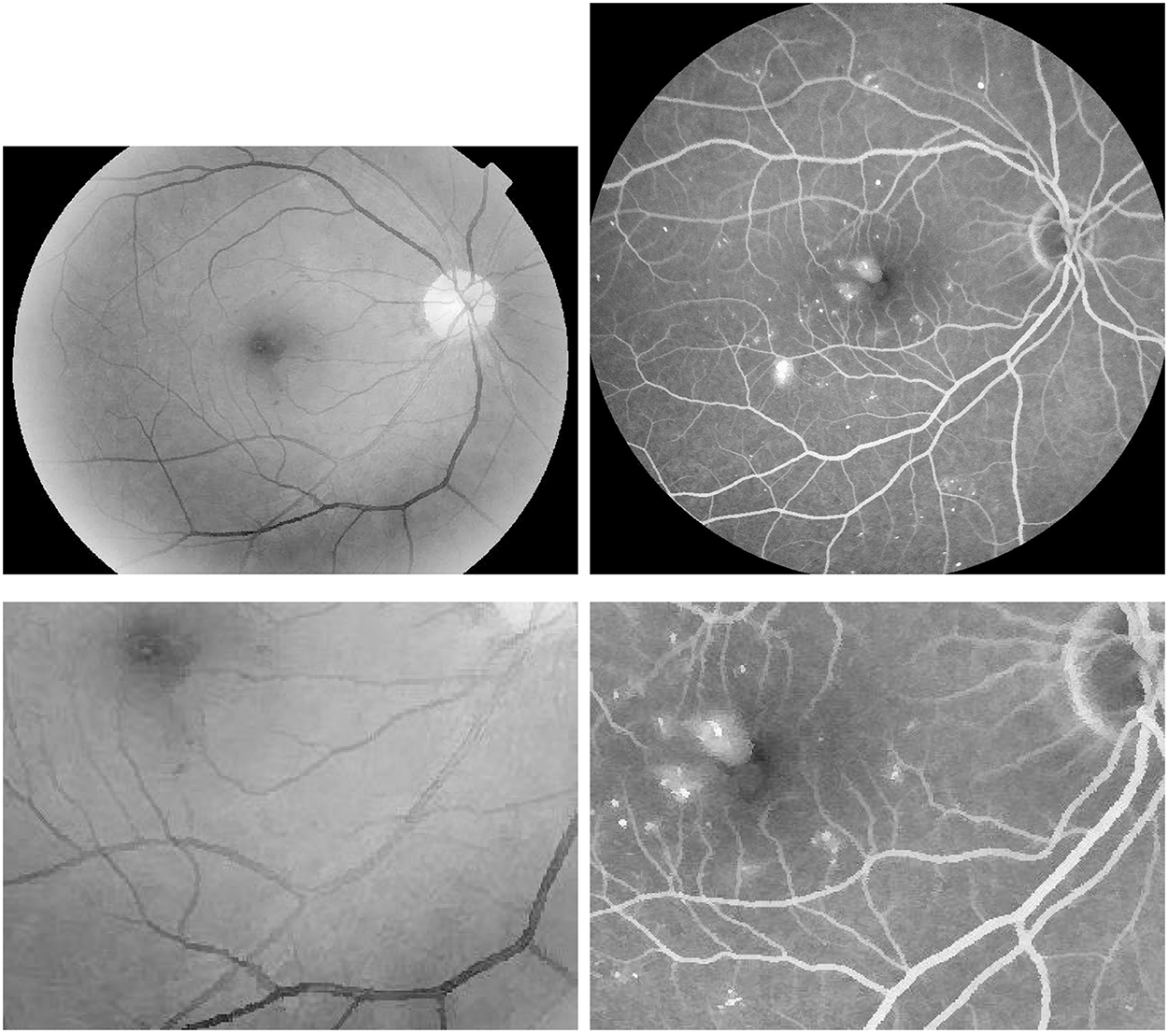
Retinal image enhancement for detection of diabetic retinopathy: top, original images; bottom, results by SF filtering after Gamma manipulation, respectively.

Finally, both retinal images are enhanced by the SF filtering (Δ*t* = 0.5, *n* = 10) after the Gamma manipulation in Fig 10. As discussed above, although the SF filtering enhances images by sharpening their edges, noise magnification and over-enhanced artifacts by the shock computing can be obviously observed. False over-enhanced details will make it difficult for a physician to identify abnormal pathologic changes correctly.

It is important to point out that the parameters in the proposed method will greatly affect the results of retinal image enhancement. For a specific system of fundus retina imaging, the parameters in the proposed method can be fixed by a certain amount of data simulations and tests.

## Conclusions (V)

In retinal image data enhancement for early detection of diabetic retinopathy, it is crucial to highlight important pathological features such as microaneurysm, bleeding and exudating spots. In this paper, a robust inverse diffusion equation is presented by combining a powerful self-similarity filtering, where the flux corrected transport technique is used to eliminate overshoots inherent in the Laplacian operation. At the same time, the self-similarity filtering not only effectively removes image noise, but also avoids noise magnification common in image enhancement methods, resulting in a robust processing of noisy and blurry retinal image data. Experimental results demonstrate that this algorithm can enhance important details of image data effectively without overshoots and noise magnification, affording an opportunity for better medical interpretation and subsequent processing.

For future research, we will further try to optimize the algorithm in the process of adaptive image enhancement according to the gray-level distribution of retinal lesions.

## Supporting Information

S1 Fig Test data in diabetic retinopathy: left, image with microaneurysms; right, image with soft exudations. (DOC)
