# Supplementary material for: Retinal Image Enhancement Using Robust Inverse Diffusion Equation and Self-Similarity Filtering
Source: PLoS One. 2016 Jul 7;11(7):e0158480. doi: 10.1371/journal.pone.0158480 (PMC4936706; doi:10.1371/journal.pone.0158480)

Test data in diabetic retinopathy: left, image with microaneurysms; right, image with soft exudations.


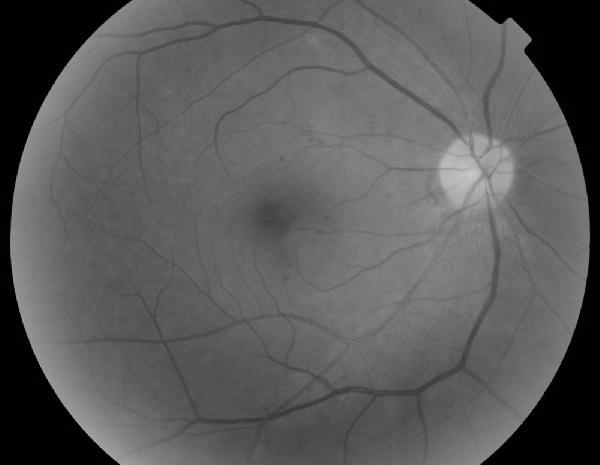

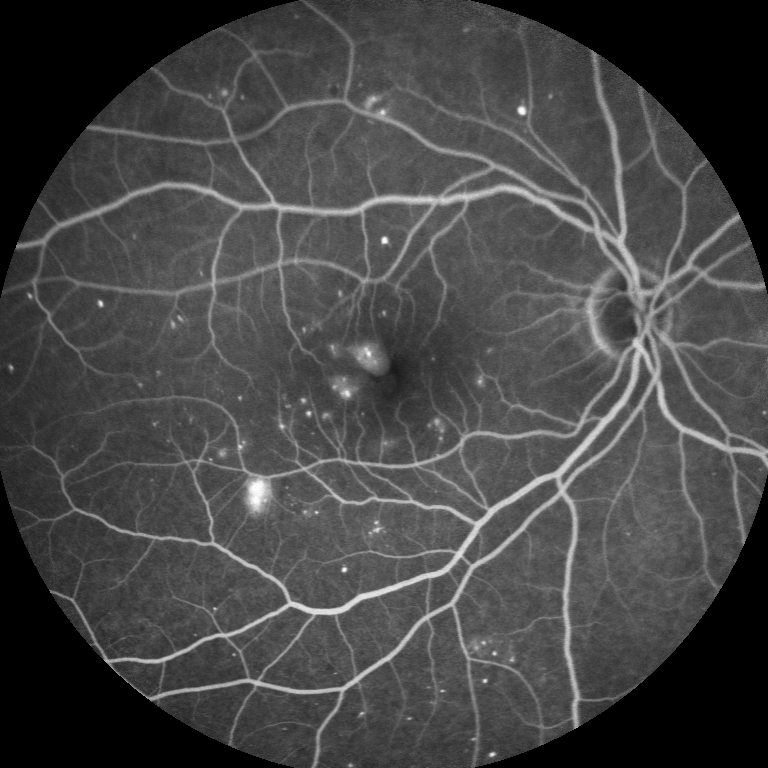

Supplement: S1 Fig — (DOC) [file pone.0158480.s001.doc]
